# 270 3-day Duration for Hospitalized Patients with Community-acquired Pneumonia: Results of a 68 Hospital Survey

**DOI:** 10.1017/ash.2026.10633

**Published:** 2026-06-23

**Authors:** Josy Lamour, Alaina Stoute, Karina Albuquerque, Maurice Policar, Laura Iavicoli, Linda Mundy

**Affiliations:** 1 NYC Health+Hospital/Elmhurst; 2 Elmhurst Hospital; 3 Elmhurst Hospital Center; 4 New York City Health and Hospitals/Elmhurst; 5 Icahn School of Medicine at Mount Sinai

## Abstract

**Background.** Clinical identification of measles prompts post-exposure prophylaxis (PEP) and surveillance. This report informs on implementation, results, facilitators and challenges associated with two measles post-exposure investigations (MPEI). Methods. Two MPEI were conducted in 2025 at a 545-bed public NYC Health+Hospitals. Measles case evaluations utilized the Identify-Isolate-Inform (I3) tool and the MPEI employed Identify-Inform-Initiate PEP-Isolate process (I4). Measles exposure was defined as a person in the Emergency Department (ED) within two hours of the index case. PEP for non-immune exposures was MMR vaccination within 72 hours (immunocompetent), measles intravenous immunoglobulin (IVIG) within six days (pregnant, immunocompromised), or notification to self-isolate for a 21- to 28-day surveillance period for non-immune persons who did not receive PEP. The exposure window was defined after security tape reviews of ED index-case entries. Epic (Verona, WI) facilitated exposure recalls; a ‘Measles rule out’ banner was uploaded to each chart. Internal and external stakeholders were informed after suspected case isolation. Recalls were conducted using standardized scripts tailored to initial responses and identified accompanying persons. MMR vaccination status was screened for in Epic and NYC vaccine registry. Descriptive analyses were calculated for MPEI. Results. Case 1 presented in Spring 2025 with airborne infection isolation room (AIIR) placement within 16 minutes. Case 2 presented in Summer 2025 with AIIR placement within 10 minutes. Each case had measles serology and nasopharyngeal swab for PCR obtained; the NYC Public Health Laboratory confirmed measles for Case 1 and Case 2 at 28- and 11-hours post-presentation, respectively. There were 145 adults and one child identified in the two MPEI. Spring MPEI identified 70 patients, 11 accompanied persons, 34 staff; Summer MPEI identified 22 patients, three accompanied persons, six staff. Fifty-nine of 146 (40%) exposures were measles immune, 30 received MMR, 3 non-immune pregnant women and one immunocompromised host received measles IVIG; 53 persons were notified to self-isolate through the surveillance period. No secondary cases of measles were identified. Discussion. Each MPEI was initiated after astute identification of a measles case. Key MPEI facilitators were an existing I3 preparedness plan and prior trainings, rapidly defined epidemiological exposure windows, leveraged use of Epic, and a structured post-exposure I4 process. Major challenges were related to uncertainty of a potential surge of cases requiring internal communications for patient flow during the recall process and for streamlined bidirectional communication with public health agencies. We acknowledge limitations of a single-site study and inherent biases of retrospective study design.

